# Transcranial Magnetic Stimulation for Patients with Exposure Therapy Resistant Obsessive-Compulsive Disorder (TETRO): Study Protocol for a Multicenter Randomized Controlled Trial

**DOI:** 10.2196/94288

**Published:** 2026-09-09

**Authors:** Tjardo S Postma, Ysbrand D van der Werf, Martijn Arns, Neeltje M Batelaan, Manou van den Berg, Judith E Bosmans, Merijn Eikelenboom, Gert-Jan Hendriks, Adriaan W Hoogendoorn, Mirjam Kampman, Désirée van Leeuwen, Iris van Oostrom, Patricia van Oppen, Alexander T Sack, Koen Schruers, Indira Tendolkar, Chris Vriend, Odile A van den Heuvel

**Affiliations:** 1 Department of Psychiatry, Amsterdam UMC Location Vrije Universiteit Amsterdam, De Boelelaan 1117, Amsterdam, North Holland, 1081HV, The Netherlands, 31 0204444444; 2 Department of Anatomy & Neurosciences, Amsterdam UMC Location Vrije Universiteit Amsterdam, Amsterdam, North Holland, The Netherlands; 3 Impulsivity & Attention program, Amsterdam Neuroscience, Amsterdam, North Holland, The Netherlands; 4 GGZ inGeest, Amsterdam, North Holland, The Netherlands; 5 Brainclinics foundation, Brainclinics, Nijmegen, Gelderland, The Netherlands; 6 Department of Psychiatry and Behavioral Sciences, Stanford University, Stanford, CA, United States; 7 Amsterdam Public Health, Amsterdam, North Holland, The Netherlands; 8 Neurocare clinics, Nijmegen, Gelderland, The Netherlands; 9 Faculty of Science, Department of Health Sciences, Vrije Universiteit Amsterdam, Amsterdam, North Holland, The Netherlands; 10 Overwaal Centre for Anxiety, OCD and PTSD, Pro Persona, Nijmegen, Gelderland, The Netherlands; 11 Behavioral Science Institute, Radboud University Nijmegen, Nijmegen, Gelderland, The Netherlands; 12 Department of Psychiatry, Radboud University Medical Center, Nijmegen, Gelderland, The Netherlands; 13 Angst Dwang en Fobie (ADF) stichting, Driebergen-Rijsenburg, The Netherlands; 14 Department of Cognitive Neuroscience, Faculty of Psychology and Neuroscience, Maastricht University, Maastricht, Limburg, The Netherlands; 15 Maastricht Brain Imaging Centre (MBIC), Maastricht University, Maastricht, Limburg, The Netherlands; 16 Brain and Nerve Centre, School for Mental Health and Neuroscience, Maastricht University Medical Centre, Maastricht, Limburg, The Netherlands; 17 Department of Psychiatry and Neuropsychology, School for Mental Health and Neuroscience, Maastricht University, Maastricht, Limburg, The Netherlands; 18 Topclinical center for anxiety disorders, OCD and PTSD, Mondriaan GGZ, Maastricht, Limburg, The Netherlands; 19 Department of Psychiatry, Donders Institute for Brain, Cognition and Behaviour, Nijmegen, Gelderland, The Netherlands

**Keywords:** rTMS, repetitive transcranial magnetic stimulation, ERP, exposure therapy and response prevention, OCD, obsessive-compulsive disorder, pre-SMA, presupplementary motor area, RCT, randomized controlled trial, neuroimaging, neuronal plasticity, cost-effectiveness analysis

## Abstract

**Background:**

Obsessive-compulsive disorder (OCD) is a disabling mental disorder, characterized by obsessions, compulsions, and substantial morbidity. Approximately 50% of adults with OCD fail to achieve satisfactory outcomes from first-line treatments, such as exposure therapy with response prevention (ERP), with or without medication. This leads to chronic social, educational, and occupational impairment. While invasive procedures such as deep brain stimulation are available for severe, treatment-refractory cases, a need remains for less invasive alternatives. Repetitive transcranial magnetic stimulation (rTMS), a noninvasive intervention, shows promise in reducing OCD symptoms. Unlike in depression, rTMS is not yet reimbursed for OCD in the Dutch healthcare system.

**Objective:**

This study examines the efficacy and cost-effectiveness of low-frequency (1Hz) rTMS targeting the presupplementary motor area (pre-SMA) compared to sham rTMS as an adjuvant treatment to ERP in adults with OCD with inadequate response to first-line treatment.

**Methods:**

A total of 250 adults with OCD will be enrolled in this multicenter randomized controlled trial. Participants will be randomly assigned to ERP combined with either active or sham 1Hz rTMS over the pre-SMA. Treatment is administered 4 times weekly for at least 5 weeks (20 rTMS-ERP sessions), with optional extension of 1 to 2 weeks, up to 28 rTMS-ERP sessions. Clinical assessments occur at baseline, weekly during treatment, posttreatment, and at 3, 6, and 12 months follow-up. Participants undergo pre- and posttreatment (functional) (MRI) scans, including a symptom provocation task. Blood sampling takes place pre- and posttreatment and at 3-month follow-up. The primary outcome is OCD severity at posttreatment, as measured by the Yale-Brown Obsessive-Compulsive Scale (Y-BOCS). Secondary outcomes include functional improvement, quality of life, and societal costs. Pretreatment symptom profiles, genotype, and brain network topology will be analyzed as predictors of response and relapse risk. Pre-to-post treatment change in blood-based and magnetic resonance (MR)-based neuroplasticity markers will help explore differential mechanisms between ERP alone and combined rTMS-ERP. We expect that the verum rTMS protocol will be cost-effective compared to sham-rTMS.

**Results:**

Recruitment started in April 2022, and as of February 2026, 201 participants have been enrolled. Posttreatment assessments are projected to be completed in December 2026, with final one-year follow-up evaluations anticipated by the end of 2027.

**Conclusions:**

To our knowledge, this study is the first adequately powered randomized controlled trial examining efficacy, cost-effectiveness, and mechanism of action of rTMS for OCD as adjuvant therapy to ERP. In case of efficacy and/or cost-effectiveness, it will pave the way for rTMS as insured health care for adults with OCD in the Netherlands, and possibly other European countries. Furthermore, this trial will provide insight into the mechanisms of treatment response to intensive ERP, with and without adjunctive rTMS, as well as potential side effects, individual variability, and long-term outcomes in adults with OCD.

## Introduction

### Background

Obsessive-compulsive disorder (OCD) is a serious and disabling mental disorder with a lifetime prevalence of 2% [1]. It is characterized by obsessions and compulsions and is associated with substantial morbidity [2]. Approximately 50% of individuals with OCD fail to achieve satisfactory outcomes from first-line treatments such as exposure therapy with response prevention (ERP) with or without a serotonergic antidepressant, resulting in chronicity and impaired social, educational, and occupational functioning [3]. In Europe, OCD accounts for an estimated 329,684 disability-adjusted life years (DALYs), corresponding to a rate of 7.9 per 10,000 population [4].

Invasive neuromodulation approaches such as deep brain stimulation (DBS) are only an option for a small and highly restricted group of severe, treatment-refractory cases. This highlights the large gap between the many adults with OCD who benefit from ERP and medication, and the few who are eligible for or actually receive DBS, underscoring the need for less invasive alternatives.

Neurodevelopmental alterations are thought to increase vulnerability to OCD [5]. In turn, the chronic course of the disorder, through repeated patterns of thought and behavior, can further shape the neural circuits underlying compulsivity [2,6]. Two nonpharmacological interventions that may help modify these brain circuits are intensive behavioral training, such as ERP, and repetitive transcranial magnetic stimulation (rTMS). Combining these 2 modalities may, through engaging synergistic neural circuitry [7], further enhance treatment effects. Despite the promising efficacy of rTMS in reducing OCD symptoms [8], it is not yet reimbursed within the Dutch health care system, unlike for treatment-resistant depression. While evidence for cost-effectiveness has been shown for deep TMS in OCD [9], the cost-effectiveness of rTMS as adjuvant treatment to ERP has, to our knowledge, yet to be evaluated. Because the present manuscript extends methods and findings from our earlier studies, several references throughout the Introduction relate to our previous work.

### rTMS

rTMS is a noninvasive neuromodulation technique that delivers repeated magnetic pulses to selectively stimulate a targeted brain region and its interconnected network [10]. Likely mechanisms underlying rTMS include changes in neuroplasticity leading to alterations in brain function and connectivity [11]. Neuroplasticity refers to the brain’s capacity for use-dependent change. There is evidence of rTMS-induced neuroplasticity at multiple levels in the nervous system, such as increases in brain-derived neurotrophic factor (BDNF) at the synapse level, increases or decreases in excitability at the neuronal level, and changes in brain activation and functional connectivity at the system level [11]. Additionally, repeated modulation of circuit-wide neuronal activity leads to structural changes in the brain, such as increases in white matter integrity [12-14] and increases in gray matter volume and cortical thickness [15,16].

Over the past 4 decades, rTMS has been used both as a research tool and a therapeutic intervention for various neurological and psychiatric conditions [17]. Interest in rTMS as a treatment for OCD has grown over the past 2 decades; the favorable safety profile and relatively minor side effects of rTMS make it an appealing therapeutic approach [18]. Various stimulation targets have been investigated, most notably the presupplementary motor area (pre-SMA) and the dorsolateral prefrontal cortex (DLPFC) [8], using both high-frequency (HF) and low-frequency (LF) protocols. Additionally, deep rTMS, a form of rTMS capable of modulating deeper cortical structures such as the anterior cingulate cortex using specialized coils, has emerged as a promising approach. Although direct comparisons between different rTMS targets and protocols in the literature have been understudied and (network) meta-analyses have shown inconclusive results, LF rTMS to the pre-SMA has been highlighted as one of 3 effective rTMS protocols in a recent meta-analysis [8].

Until recently, rTMS has been primarily studied, compared to sham treatment, as a stand-alone treatment in OCD. Current research nevertheless increasingly suggests that combining rTMS with established therapies may produce synergistic effects and improve outcomes [19,20]. The decision to study combined rTMS and ERP was based on the clinical and theoretical premise that neither intervention alone is sufficient for many patients with severe and chronic OCD. Although ERP remains the gold standard treatment, a substantial proportion of patients either discontinue treatment early due to difficulty tolerating the exposure exercises, derive only partial benefit, or continue experiencing clinically significant symptoms following treatment [3]. At the same time, while meta-analyses support the efficacy of rTMS compared to sham stimulation in OCD, the observed effects are generally modest, and their durability remains uncertain, with follow-up periods in many studies limited to several weeks [21]. Therefore, the most clinically relevant question is not whether rTMS can function in isolation, but whether it can provide additional therapeutic value when integrated into an evidence-based treatment framework.

This rationale is further supported by emerging evidence suggesting that the effects of rTMS are state-dependent [22], meaning that the cognitive and behavioral context during stimulation may influence treatment efficacy. In OCD specifically, this may be particularly relevant given the rigidity and chronicity of compulsive behaviors. ERP and rTMS likely target overlapping but distinct mechanisms: ERP promotes inhibitory learning, cognitive flexibility, and behavioral adaptation through exposure [23], whereas rTMS may facilitate cortical excitability and network plasticity [11]. Combining these approaches may therefore create conditions that are more favorable for therapeutic learning and more durable symptom change than either intervention alone [20,24]. This approach is also consistent with findings from other psychiatric disorders. In depression, there is evidence that psychotherapy and noninvasive brain stimulation could outperform neuromodulation monotherapy [25]. In anxiety disorders, active rTMS combined with psychotherapy showed significantly greater symptom improvement compared to sham rTMS [20]. Although evidence for combined treatment in OCD remains limited, these findings provided an important rationale for investigating this strategy in a treatment-resistant OCD population. An additional advantage of this design was that all participants received an active and potentially beneficial intervention, as prior insufficient response to ERP does not necessarily imply that more intensive ERP-based approaches are ineffective [26]. This may also positively influence recruitment and reduce dropout in this severely affected patient population. In summary, the use of LF rTMS over the pre-SMA alongside ERP therapy reflects the direction of clinical and ongoing experimental treatment in the field.

### Cost-Effectiveness

No national or international studies have to date evaluated the cost-effectiveness of rTMS as adjuvant treatment to ERP for OCD. Recently, rTMS has become an established and evidence-based treatment for treatment-resistant depression [27]. It has been integrated into national treatment guidelines and is reimbursed by an increasing number of health care systems, including the Dutch healthcare system.

Several studies have found rTMS to be cost-effective compared to sham treatment in the context of major depressive disorder (MDD). Specifically, 3 economic evaluations concluded that rTMS becomes cost-effective after one failed trial of antidepressants [27-29]. Because MDD and OCD are both chronic psychiatric disorders that share a similar burden on the health care system, and their type and efficacy of first-line treatments often overlap, these findings suggest that rTMS can similarly provide significant savings when used in individuals with OCD who have not benefited sufficiently from first-line treatments.

OCD is typically characterized by a chronic course, with symptoms that tend to persist over many years. Findings from the Netherlands OCD Association (NOCDA) study, one of the largest longitudinal studies on OCD to date, illustrate this persistence, showing that 61.7% of participants already had chronic OCD, defined as at least 2 years of moderately severe symptoms, at enrollment [30]. Further analyses have indicated that chronic symptoms at baseline strongly predict continued chronicity after a 2-year naturalistic follow-up [31]. This is consistent with findings from a US study showing that failure to achieve remission over a 5-year period was a strong predictor of long-term chronicity, and that even partial remission was associated with a high risk of relapse [32]. Additionally, OCD severity has been linked to increased occupational disability and significant socioeconomic burden [33]. These findings underscore the importance of maximizing the effectiveness of treatment.

Although rTMS may initially be more expensive than standard care, it is expected to improve recovery rates and enhance quality of life (QoL) for people with OCD. In the long term, these improvements could shorten treatment duration, increase social and occupational functioning, and ultimately lower both health care and lost productivity costs. Repetitive TMS might also serve as a less invasive and less costly alternative to DBS. The primary added value of the combined usage of ERP with rTMS lies in its potential to reduce chronicity, improve level of functioning and QoL, and lower the long-term costs associated with health care use and lost productivity.

### Durability

As of 2025, evidence on the long-term outcomes of rTMS for OCD remains limited. To address this gap, our study includes follow-up assessments at 3, 6, and 12 months posttreatment. This is particularly important given the expectation that potential cost savings are likely to emerge only after a 12-month follow-up period. These data will contribute to a better understanding of the durability of treatment effects over time.

### Aim

Our aim is to bridge the treatment gap between the approximately 50% of people with OCD who benefit from standard interventions (ERP with or without SSRIs) and the small minority who undergo neurosurgical procedures. To this end, we propose a noninvasive alternative: rTMS as an augmentative strategy to enhance the efficacy of intensive ERP. This study will evaluate the added value of low-frequency (1Hz) rTMS targeting the pre-SMA, compared to sham stimulation, in individuals with OCD who have shown limited or no durable response to ERP alone or in combination with pharmacotherapy. Second, we aim to improve our understanding of the mechanisms underlying rTMS by investigating the effects of ERP and rTMS across multiple levels using multimodal neuroimaging and blood-based markers of neuroplasticity. In addition, we aim to identify pretreatment symptom profiles, genetic factors, and brain network characteristics associated with treatment response and risk of relapse.

### Hypotheses

We hypothesize that rTMS combined with ERP will be more effective and cost-effective than sham-rTMS combined with ERP. However, we also expect clinically meaningful symptom reduction in the sham condition, as all participants receive intensive ERP at specialized OCD centers. Regarding neuroplasticity outcomes, we expect both treatment groups to show changes in OCD-relevant brain networks associated with cognitive control and fronto-limbic connectivity, reflecting treatment-related neural plasticity. We further hypothesize that these effects will be stronger in the active rTMS group, particularly in sensorimotor networks following pre-SMA stimulation. In addition, symptom improvement is expected to be associated with changes in BDNF-related molecular markers, with greater effects in the active rTMS condition.

## Methods

### Study Design

This study is a 2-arm randomized controlled trial (RCT) with an active adjuvant rTMS treatment with ERP delivered 4 times per week; the control condition involves sham rTMS combined with ERP. Treatment duration is adaptive, based on clinical improvement, and consists of a minimum of 20, and a maximum of 28 combined rTMS-ERP sessions. Participants undergo a baseline MRI scan session to analyze pre-to-posttreatment changes in brain structure and function and for prediction analyses of response and relapse. Blood sampling is performed at baseline, directly posttreatment, and at 3 months follow-up. Participants, ERP therapists, and clinical assessors remain blind to treatment conditions during treatment and follow-up. Outcome measures are assessed at baseline, posttreatment, and at 3, 6, and 12 months follow-up. A subset of these measures are assessed weekly during treatment (see outcome measures). Neuronavigation is used to record coil placement during the 1st, 10th, and 20th session.

### Setting of the Study

This multicenter trial is carried out in specialized mental health care facilities across the Netherlands. Participants are recruited through multiple channels: outpatient clinics with extensive experience in the treatment of OCD, self-referral, and through the patient organization for Anxiety and OCD. The patient organization was also actively involved in the design of the trial, and one of their representative lived experience researchers participates in the monthly research meetings to support ongoing collaboration and alignment with patient perspectives. The 5 participating sites geographically cover the country, enabling treatment in the catchment area of the participants’ home. These 5 sites are located in Amsterdam (Amsterdam UMC/GGZ inGeest), Nijmegen (Radboudumc/ProPersona, with MRI scans at Donders Center of Cognitive Neuroimaging), Maastricht (Maastricht UMC+/Mondriaan groep, with MRI scans at Scannexus), and Eindhoven and Groningen (2 sites of neurocare clinics). Participants from Eindhoven and Groningen undergo MRI scans in either Amsterdam or Nijmegen, based on their preference. The 5 centers aim to contribute equally to patient inclusion (between 35‐60 participants per site). The following rTMS machines are used in this trial: Magstim rapid2 (Amsterdam UMC, Radboudumc, Maastricht UMC+) and Deymed XT 35 (neurocare Eindhoven and Groningen).

### Participants

We aim to include 250 adults with OCD (aged 18 y and older) who have not responded to at least 8 sessions of state-of-the-art ERP. The inclusion and exclusion criteria are designed to target individuals whose symptoms are insufficiently managed by standard treatments (ERP with or without serotonergic antidepressants) but who have not yet progressed to more invasive interventions such as DBS. Eligibility requires a primary diagnosis of OCD according to the *Diagnostic and Statistical Manual of Mental Disorders, Fifth Edition* (*DSM-5*) criteria, with symptom severity in the moderate to severe range as indicated by a Yale-Brown Obsessive Compulsive Scale (Y-BOCS)-I score of 16 or higher [34]. Exclusion criteria consist of recent changes in psychotropic medication (less than 8 wk before the trial), hoarding as the main symptom dimension, presence of a psychotic disorder, bipolar disorder, autism spectrum disorder (when this dominates the clinical profile, ie, is diagnosed as the primary disorder), substance use disorder, and current suicidal ideation with intent. Participants are also excluded if they are unable to safely undergo TMS treatment due to contraindications such as metallic implanted devices (eg, pacemakers and cochlear implants), epilepsy, any neurological disorder that causes a lesion to the brain and increased seizure risk (such as multiple sclerosis, previous serious head trauma, and stroke), pregnancy, chronic benzodiazepine use, or had rTMS in the past (for blinding reasons). Participants with severe claustrophobia who were unable to undergo MRI scanning were still eligible to participate in the trial.

### Study Procedures

**Figure 1.**
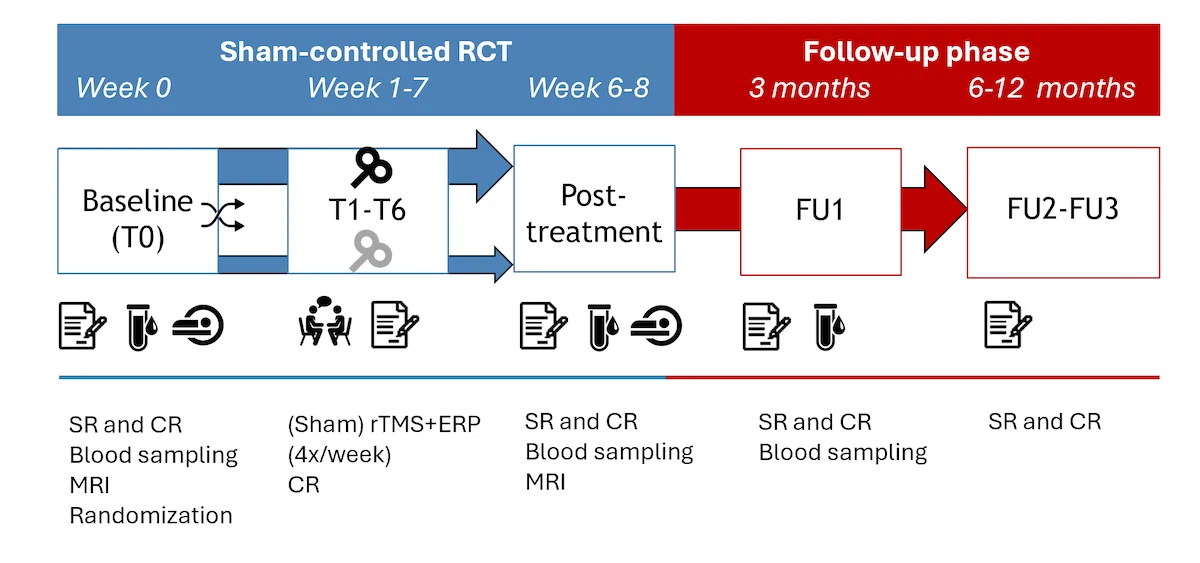
An overview of the study timeline. CR: clinician-rated questionnaires; ERP: exposure and response prevention; FU: follow-up; RCT: randomized controlled trial; rTMS: repetitive transcranial magnetic stimulation; SR: self-reports.

Information about the study is provided to eligible participants, after which they are contacted again after at least 7 days to discuss participation and answer remaining questions. After screening for eligibility and signing informed consent, participants are invited for a baseline measurement, an MRI scan session, and blood sampling (Figure 1). Different outcome measures are assessed at different time points: pretreatment, during treatment, posttreatment, and at 3, 6, and 12 months follow-up. For an overview of the study assessments, see Table 1. Subjects can leave the study at any time for any reason if they wish to do so without any consequences. In addition to clinical outcomes, cost-effectiveness is evaluated over the 12-month follow-up period.

**Table 1.** Primary and secondary outcomes and time points.

Clinical		Cost-effectiveness		Neuroplasticity	
Y-BOCS^a^ I	Pre- and posttreatment	iPCQ^b^	Pre- and posttreatment, FU1-FU3	MRI (structural and functional)	Pre- and posttreatment
Response	Posttreatment, FU1-FU3	iMCQ^c^	Pre- and posttreatment, FU1-FU3	GWASd^d^	Pretreatment
Remission	Posttreatment, FU1-FU3	EQ-5D-5L	Pre- and posttreatment, FU1-FU3	BDNF^e^ and pro-BDNF	Pre- and posttreatment, FU1
Y-BOCS II	Pre- and posttreatment, weekly during treatment, FU1-FU3	—^l^	—	NDEV^f^ analysis	Pre- and posttreatment, FU1
CGI^g^	Pre- and posttreatment, FU1-FU3	—	—	—	—
BDI^h^ and VAS^j^ mood	Pre- and posttreatment, FU1-FU3	—	—	—	—
BAI^i^ and VAS anxiety	Pre- and posttreatment, FU1-FU3	—	—	—	—
YGTSS^k^ (in case of tics)	Pre- and posttreatment, FU1-FU3	—	—	—	—
Side effects	Weekly during treatment	—	—	—	—

a Y-BOCS: Yale-Brown Obsessive Compulsive Scale.

b iPCQ: IMTA Productivity Cost Questionnaire.

c iMCQ: iMTA Medical Consumption Questionnaire.

d GWAS: Genome-wide Association Study.

e BDNF: brain-derived neurotrophic factor.

f NDEV: neuron-derived extracellular vesicle.

g CGI: clinical global impression.

h BDI: Beck depression inventory.

i BAI: Beck anxiety inventory.

j VAS: Visual Analog Scale.

k YGTSS: Yale Global Tic Severity Scale.

l Not applicable

### Image Acquisition and Preprocessing

Within 2 weeks prior to treatment and within 2-weeks posttreatment, whole brain structural and functional MRI images are acquired. All sites use a 3 Tesla Siemens (Erlangen, Germany) scanner: type VIDA 3T in Amsterdam UMC, and type Magnetom Prisma in Maastricht UMC+ and Radboudumc. Sequences included in the pre- and posttreatment scans are:

1. High-resolution T1-weighted structural scan (T1), using a 3D T1-weighted structural magnetization-prepared rapid acquisition gradient-echo (MPRAGE): repetition time (TR) 2300 ms, echo time (TE) 2.26 ms, 192 slices, voxel size 1×1×1 mm, and flip angle 8 degrees.
2. Resting-state functional MRI data are collected over a 10-minute period while participants maintain visual fixation on a black cross presented against a white background. Imaging is performed using T2*-weighted echo-planar images (TR 2270 ms, TE 28 ms, flip angle 80 degrees, 3.3 × 3.3 mm² in-plane resolution, 42 sequentially ascending slices of 3 mm with 0.3 mm gap, 268 volumes).
3. Functional MRI (fMRI) during a symptom provocation task (SPT). This task involves the presentation of general fear-related and OCD-specific images [35]. The fear- and OCD-related stimuli were adapted from de Wit et al [36]. Scrambled versions of the aforementioned pictures are used as neutral conditions (T2*-weighted echo-planar images with multiband factor 2, TR 1800 ms, TE 28 ms, flip angle 70 degrees, 3.3 × 3.3 mm² in-plane resolution, 64 sequentially ascending slices of 2 mm with 0.2 mm gap, 400 volumes). Scans with opposite phase-encoding directions were acquired to correct for susceptibility-induced distortions
4. 3D FLAIR (TR 5000 ms, TE 386 ms, 160 slices, with resolution 1×1×1 mm, and flip angle 120)
5. Diffusion-weighted image (DWI) is acquired with a multiband (MB=3) multishell single-spin-echo echo-planar imaging sequence with 135 diffusion-weighted directions (22 b=1000 s/mm2 , 44 b=2000 s/mm2 and 66 b=3000 s/mm2) and 13 nondiffusion-weighted volumes (b=0 s/mm2). A scan with opposite phase-encoding directions was acquired to correct for susceptibility-induced distortions.

Following the scan, participants rate their anxiety and tension in response to the symptom provocation stimuli using a 1‐9 Visual Analog Scale (VAS). Separately, VAS scores (0‐100) are used to assess overall tension before and after resting-state and symptom provocation scans. Total scanning time is 60 minutes.

### Randomization

Participants are randomized in a 2:1 ratio to one of 2 treatment arms, with twice as many participants allocated to receive active stimulation of the pre-SMA compared to sham rTMS. This unequal allocation was chosen to maximize the number of participants receiving the active intervention and to enhance recruitment by increasing the probability of assignment to active treatment.

Randomization is stratified by study sites to ensure balanced allocation across centers. Randomization sequences are generated using Castor EDC. Each site implements block randomization using randomly permuted blocks of size 6 to maintain allocation balance within each center and minimize group size imbalances that may arise due to site-specific factors, such as varying levels of experience with rTMS.

### Intervention

Treatment consists of 4 sessions per week in which each rTMS session is immediately followed by a 90-minute ERP session. This offers the logistical benefit of catching up on a missed treatment during a treatment week. The treatment follows an adaptive design, offering a minimum of 20 sessions to all participants with a possible extension of 4 or 8 sessions for participants who continue to show improvement and/or are motivated to continue therapy (see decision rules below) for optimal treatment effect. Change in symptoms is measured weekly during the treatment by administration of the Y-BOCS-II [37] to obtain a severity score after every 4 individual sessions. After 4 weeks of combined treatment, the process of the treatment is evaluated with the patient and one of the clinicians to decide if treatment should be continued after the 5th week. The decision process on (dis)continuation always follows shared decision making and is independent of treatment allocation; in case of doubt, the case is to be discussed in the weekly indication & treatment meeting. Participants who, after clinical evaluation, feel that they can still benefit from treatment can continue. If clinicians and participants agree that treatment will not be beneficial anymore or if participants worsen during treatment, treatment is discontinued. At the end of week 6, we again monitor progress using the same criteria, allowing participants who continue to show steady (partial) response the opportunity to undergo a seventh week of treatment, amounting to a maximum of 28 sessions per participant. This design allows participants who are experiencing protracted and slowly accruing benefit from the repeated combined sessions to maximize their treatment effect; and it accounts for the slow and linear benefit of repeated rTMS sessions that has been observed in adults with OCD, who still improve at or after 20 sessions [38,39]. Additionally, it allows for participants not benefiting enough from the treatment to exit the study and prevent unnecessary burden.

### rTMS Parameters

Previous meta-analyses have shown conflicting and inconclusive results about the efficacy of different stimulation targets and protocols in OCD [40-42]. We based our choice of stimulation location, intensity, duration, and frequency on recommendations from international guidelines and following the pairwise and network meta-analysis that our group carried out prior to the start of the study [8]. Treatment thus combines low-frequency (1-Hz) rTMS over the pre-SMA with ERP, administered immediately after the rTMS session. The intensity of stimulation is set at 110% of resting motor threshold (RMT), determined in one session a week before the start of the treatment, by trained experimenters through visual observation of muscle activation in the first dorsal interosseous muscle of the right hand, in response to handheld single-pulse stimulation. RMT is defined as 2 motor responses in a series of 4 consecutive single-pulse stimulations. In the verum rTMS condition, we deliver 1500 continuous 1-Hz pulses to the pre-SMA, that is, 25 minutes of stimulation. This protocol allows us to obtain a maximum stimulation effect while conforming to internationally published safety guidelines [18].

### TMS Coil Placement and Recording of Sessions

The positioning of the TMS coil on the scalp is based on the EEG 10‐20 coordinate system. To localize the pre-SMA, the midpoint between the tragi is first identified and marked. Using this reference point, the nasion-inion distance is measured with a flexible measuring tape. From the nasion, 35% of the total nasion-inion distance is measured along the curved path of the tape, ensuring it passes through the previously marked midpoint between the tragi. The location corresponding to this 35% point (15% anterior of the vertex) is marked on the scalp as the pre-SMA target site. The coil is placed on the marker with the handle pointing in the posterior direction.

Precision of the rTMS coil positioning is recorded using neuronavigation during the 1st, 10th, and 20th sessions [43], available in all collaborating centers. The procedure of neuronavigation requires the use of a high-resolution T1-weighted MRI scan, registered to the participant’s head upon co-registering visible landmarks on the head of the participant with landmarks on the MRI scan. The use of neuronavigation software allows us to record the location of every single pulse of stimulation throughout the treatment session and for post-hoc verification of the precise stimulation location in the participant’s MRI in native space, and at group level in standard space.

This approach will allow us to post-hoc map individualized resting-state functional connectivity-based coordinates (or even multimodal MRI-based coordinates) relative to the actually stimulated coordinate in order to relate treatment success to stimulation location (distance between actual and optimal fMRI-based coordinates). This approach will serve to inform us on the added value of MRI-based individualized targeting using neuronavigation versus nonneuronavigated rTMS treatments. This is relevant for future use of MRI-based neuronavigation in daily clinical practice.

### Comparator

The comparison group receives 4 days per week sham-rTMS (using a sham coil) followed by 90 minutes of ERP similar to the other treatment arm. Sham-rTMS delivers pulses at an intensity that is insufficient to affect brain activity but still mimics the sensation on the scalp. As a result, it is considered a null treatment, with any clinical effects in this group attributed to ERP alone. Sham treatment is performed over the same location, that is, pre-SMA, to further ensure blinding of the participant to the treatment condition. Additionally, the other rTMS parameters, 1500 continuous 1-Hz pulses at 110% of the RMT, are identical to those used in the active (verum) condition.

### Co-Intervention: Exposure in Vivo With Response Prevention (ERP)

Prior to the first rTMS-ERP treatment session, 2 ERP preparation sessions without TMS are planned. These sessions are used to motivate participants, reestablish the working mechanisms of ERP, and achieve commitment for active participation in exposure sessions and homework. In addition, participants are helped to define their treatment goals and to link these goals to specific exposure exercises, which can be practiced in either supervised exposure sessions following TMS or at home in unsupervised exposure exercises. ERP is provided according to standard treatment protocols for OCD. Sessions last 90 minutes, which is slightly longer than usual, permitting ERP exercises to take place beyond the confines of the therapist’s office. For this study, a treatment manual has been developed (largely based on the protocol used during the previous TIPICCO trial [19]), consisting of practical information, the time schedule, the agenda to be used in all sessions, and all forms that participants fill in during the treatment (including, for example, those for goals and exercises). Each ERP session follows a similar structure, including a reflection on the prior treatment session, discussing homework exercises, and registering whether homework has been conducted according to plan. Early in the session, clinician-supervised exposure commences in the clinic or outside, depending on the exercise. Clinicians motivate the participant, safeguard against subtle forms of avoidance behavior, and question the participant about the dysfunctional cognition (ie, the prediction the participant expects to occur). When possible, a more realistic alternative cognition is formulated. At the end of the exposure session, homework exercises are planned. ERP is provided by trained and well-supervised clinicians with varying experience and background (registered health care and clinical psychologists, psychologists at entry-level, resident psychiatrists, and behavioral therapy assistants). The clinicians have supervision and intervision every 2 weeks to share experience, discuss exposure exercises, and stagnation in the therapy. Multiple clinicians take responsibility for the treatment of one patient. Doing so enables a 4/wk treatment schedule, decreases the impact of clinician-variables on outcome, and, according to our experience in the TIPICCO-trial, facilitates a strict focus on the exposure during treatment sessions. At the end of the combined rTMS-ERP treatment (after 20, 24, or 28 sessions; see for details adaptive design above), 2 relapse-prevention sessions are scheduled. In these sessions, the possibility of relapse is discussed, and a personalized relapse prevention plan is made. Such a plan is advised in treatment guidelines, helping the participant to recognize early signs of relapse and to take action if necessary. Subsequently, we perform weekly telephone calls for 4 weeks to stimulate continued self-exposure in daily life. After these 4 weeks of self-exposure, we finish with an evaluation session. During the subsequent 1-year naturalistic follow-up phase, participants are allowed to participate in new treatments (but not rTMS) if necessary.

### Blood-Based Biomarkers of Neuroplasticity, Genetics and Multiomics Integration

Blood samples are collected at baseline, posttreatment, and 3-month follow-up in Amsterdam, Nijmegen, and Maastricht. Participants from other centers (Groningen and Eindhoven) are referred to the nearest facility for sampling. After centrifugation, the material is stored at −80 °C following standard operating procedures to ensure uniform handling across sites. Blood-based biomarkers will support identification of neurobiological mechanisms of treatment response and potential predictive markers. The analysis will include:

1. Genome-wide association study (GWAS): baseline DNA will be isolated from whole blood using standard extraction kits. Genotyping will be conducted with a high-density SNP array, followed by quality control and imputation to a reference panel. Genetic association models will examine baseline variants as predictors of proteomic profiles and clinical outcomes. Polygenic risk scores for OCD and related phenotypes will also be derived.
2. Olink proteomics: plasma samples will be analyzed with Olink panels. Samples from each participant (baseline, posttreatment, and follow-up) will be placed on the same plate to minimize batch variation. A pooled plasma control will be included on each plate to support normalization across assays.
3. SIMOA BDNF isoform quantification: concentrations of mature and pro-BDNF will be measured separately using validated Quanterix SIMOA plasma assays. These analyses will provide isoform-specific validation of Olink findings and clarify treatment-related changes.
4. Exploratory neuron-derived extracellular vesicle (NDEV) analysis: in a subset of 50 participants (150 samples; half receiving real-rTMS and half sham), neuron-derived extracellular vesicles will be isolated via immunocapture. Vesicle lysates will be digested and analyzed using parallel reaction monitoring mass spectrometry targeting BDNF, TrkB, and downstream signaling proteins (eg, ERK, AKT, and CREB). Stable isotope-labeled peptides will enable absolute quantification and evaluate whether peripheral proteomic changes mirror brain-derived molecular signatures.

### Outcome Measures

Primary outcome and primary endpoint:

1. The primary outcome is the severity of OCD as measured by the Yale-Brown Obsessive-Compulsive Scale I (Y-BOCS-I) [34]. The primary endpoint is the difference between the active and sham intervention groups in posttreatment severity of OCD, estimated using an analysis of covariance (ANCOVA) model that adjusts for pretreatment severity of OCD. The intervention effect will also be reported as a standardized mean difference.

Secondary outcomes - clinical:

1. Response (=35% reduction on Y-BOCS I) [44].
2. Remission (Y-BOCS I<12) [44].
3. The severity of OCD, as measured using the newer version of the Yale-Brown Obsessive-Compulsive Scale - Y-BOCS II [37,45]. A combined version of the Y-BOCS I and II is administered pretreatment and posttreatment. Additionally, Y-BOCS-II severity scores are collected weekly during treatment and at all follow-up sessions, enabling comparison between the 2 versions and potentially supporting further implementation of the newer Y-BOCS II.
4. The clinical global impression (CGI) severity scale [46].
5. Comorbid depression symptoms, measured using the Beck Depression Inventory (BDI) at baseline, posttreatment, and follow-up [47]. In addition, we administer a VAS for depression at these same time points, plus every week during treatment, to monitor treatment response on mood. The simultaneous administration of VAS and BDI at baseline and posttreatment will allow us to scale and validate the VAS relative to the BDI, while offering a much quicker and simpler assessment for the participants.
6. Anxiety, measured using the Beck Anxiety Inventory (BAI) and a VAS, following the same procedure and rationale as for depression [48].
7. For participants with comorbid tics, as identified using the Yale Global Tic Severity Scale (YGTSS) [49], tic severity will be assessed using the same scale.
8. Tolerability of the treatment and side effects, using an in-house questionnaire developed as part of our previous TIPICCO trial [19].

Secondary outcomes - cost-effectiveness

1. Societal costs will be measured using the iMTA Productivity Cost Questionnaire (iPCQ) [50] and the iMTA Medical Consumption Questionnaire (iMCQ) [51].
2. QoL (EQ-5D-5L) [52]. Quality-adjusted life-years (QALYs) are the most important outcome measure for health care decision makers when deciding about the reimbursement of health care interventions.

Secondary outcomes - neuroplasticity:

1. Blood sampling at baseline, directly posttreatment, and at 3-month follow-up is performed to establish markers of treatment-induced neuroplasticity and predictors of treatment response.
2. Pre- and posttreatment MRI scans to analyze ERP and rTMS-induced effects on multiple levels in the brain, using innovative multimodal neuroimaging.
3. Pretreatment structural and functional MRI scans to assess brain structure and network activation and connectivity to predict treatment response.

Exploratory variables:

1. Patient adherence to treatment protocol, as measured using the Patient Exposure and Response Prevention Adherence Scale (PEAS; [53]) and its relationship to treatment outcome. This scale is carried out by the therapists as part of usual treatment.
2. Difference between responders and nonresponders on circadian rhythm and other sleep disorders at baseline as defined by the Holland Sleep Disorders Questionnaire (HSDQ), as a possible prognostic marker for response [54,55].
3. The relationship of demographic and clinical variables (gender, age, age of onset, medication status), preexisting comorbidities (ie, comorbid tics, depression, anxiety, and autism), and treatment history to the treatment outcome. A simplified Dutch version of the Mini-International Neuropsychiatric Interview (MINI) for *DSM-5*-diagnoses is used to confirm OCD diagnosis and establish current comorbidity [56]. To specifically evaluate symptoms of comorbid autism spectrum disorder, the Dutch version of the Autism Spectrum Questionnaire (AQ-NL) [57] is administered.
4. The Y-BOCS-II symptom checklist [37] is used to categorize symptom dimensions and examine treatment effects and underlying mechanisms in different OCD subgroups.
5. Measuring the exact stimulation location as ascertained and recorded by neuronavigation on 3 separate sessions (session 1, 10, and 20) will allow us to map the variation in achieved location in relation to treatment outcome.
6. Variation in treatment expectancy (7-item credibility and expectancy questionnaire) [58] and blinding success (1-item question “in which condition do you think you were?”) in relation to treatment outcome.

### Blinding

Participants, ERP therapists, and those carrying out clinical and cognitive assessments are blinded to treatment conditions. The rTMS treatment administrators are not blinded due to the technical nature of this treatment; however, they are instructed not to disclose treatment allocation to participants, therapists, or blinded assessors.

### Sample Size Calculations

Meta-analyses [41,42] suggest an average effect size of 0.71‐0.79 (Hedges’ g) for the effect of rTMS as monotherapy. Our own meta-analysis [8] showed a lower Hedges’ g of 0.50. We use rTMS in addition to intensive 4-week daily ERP, and we therefore conservatively lowered the expected effect size to 0.4, in line with, for example, the augmentative effect of antipsychotics to either SSRI or cognitive behavioral therapy [59]. The effect size 0.4 corresponds to a pre-post intervention decrease in Y-BOCS I score of 4 points, which we consider a minimal clinically important difference (MCID). Our design uses a 2:1 randomization across the 2 conditions, with twice as many participants in the verum rTMS group. Using a significance level of alpha=.05, a 2-tailed comparison between means, and an estimated power of 0.8, the study requires 150 participants in the verum-rTMS arm and 75 in the sham-rTMS arm. Assuming a 10% dropout rate, we will include (225/0.9=) 250 participants. Participants are considered dropouts if they complete less than 60% of the minimum required 20 rTMS+ERP sessions, corresponding to fewer than 12 sessions. We apply stratified block randomization, with study center as the stratification factor, to ensure balanced allocation of conditions across centers. Although we will take the stratified design into account when analyzing the data by using it as a covariate, we will ignore its effect on the required sample size [60].

### Statistical Analyses

All statistical analyses for testing efficacy and cost-effectiveness will be done according to the intention-to-treat principle. Additionally, neuroplasticity will be analyzed based on the per-protocol sample, using a minimum number of 12 rTMS-ERP sessions as the threshold for adequate treatment. The significance level will be set at alpha=.05, 2-tailed. Safety data (eg, side effects and serious adverse events) are collected and summarized in the study results. A description of the analyses for the primary, secondary, and exploratory objectives is provided below.

#### Primary Study Parameter

The primary study parameter is the posttreatment effect of the intervention (verum-rTMS vs sham-rTMS) on the primary outcome measure (Y-BOCS I). This effect will be estimated using an ANCOVA model adjusting for baseline Y-BOCS I scores.

#### Secondary Study Parameters- Clinical

Of the clinical secondary outcomes, response (≥35% reduction in Y-BOCS I) and remission (Y-BOCS I ≤12) will be investigated using generalized linear mixed models (LMM, mixed-effects logistic regression). Pre-to-post changes in Y-BOCS II, CGI score, QoL, depression, tics, and anxiety will be tested using LMM analysis for repeated measures. The model will include fixed effects for time, study center, and the group-by-time interaction, but not for the group indicator alone, in order to adjust for potential baseline differences in the outcome variable. The group-by-time interaction term represents the between-group intervention effect and therefore constitutes the primary parameter of interest. Tolerability and side effects will be reported as absolute numbers and described qualitatively.

#### Secondary Study Parameters - Cost-Effectiveness

Cost categories that will be included are: (1) health care costs (primary and secondary care, complementary care, and home care); (2) lost productivity costs (absenteeism from paid and unpaid work, and presenteeism); and (3) participant costs (informal care and other care services paid for by participants themselves). For the valuation of health care usage, lost productivity, and informal care, Dutch standard costs from the Dutch costing guidelines [61] will be used. Medication use will be valued using prices of the Royal Dutch Society for Pharmacy. Patient and family costs other than informal care will be valued using self-reported prices. For the valuation of absenteeism from paid work, the friction cost approach will be used. We will estimate the cost-effectiveness of rTMS compared to sham rTMS after 12 months of follow-up from a societal perspective. The following effect measures will be included in the economic evaluation: (1) severity of OCD symptoms (Y-BOCS), (2) quality-adjusted life-years (EQ-5D-5L with Dutch reference values [52]). Linear regression analyses will be used to estimate cost and effect differences between both conditions while adjusting for confounders if necessary. Incremental cost-effectiveness ratios (ICERs) will be calculated by dividing the difference in the mean total costs between the treatment groups by the difference in mean effects between the treatment groups. Bias-corrected and accelerated bootstrapping with 5000 replications will be used to estimate 95% CIs around the cost differences and statistical uncertainty surrounding the ICERs. Uncertainty surrounding the ICERs will be graphically presented on cost-effectiveness planes. Cost-effectiveness acceptability curves will also be estimated, showing the probability that the intervention is cost-effective in comparison with control for a range of different ceiling ratios, thereby showing decision uncertainty [62].

#### Secondary Study Parameters - Neuroplasticity

We will first examine the effects of ERP and rTMS across multiple levels, using both blood-based and neuroimaging markers of neuroplasticity. For the neuroimaging component, we will use multimodal techniques, including both structural and functional MRI scans acquired at baseline and posttreatment. Data will be analyzed using hypothesis-driven Bayesian region of interest models and exploratory general linear model-based whole-brain analyses. All scans will undergo appropriate preprocessing using established open-source neuroimaging pipelines (eg, fmriprep [63] and mrtrix3 [64]). Structural and functional imaging data at baseline will also be examined as potential predictors of treatment outcome.

For the biomarker component, we will analyze genomic, proteomic, and extracellular vesicle–derived measures collected at baseline, posttreatment, and follow-up. Analyses will combine genome-wide and targeted biomarker data using both Bayesian and conventional statistical approaches. Longitudinal changes in proteomic and circulating biomarker levels will be modeled using linear mixed-effects models to account for repeated measures. Genetic association and polygenic risk score analyses will assess baseline genetic variation as a predictor of molecular profiles and clinical outcomes.

#### Exploratory Moderators and Mediators of Treatment Response

The exploratory outcomes and parameters that will be investigated as modifiers of treatment response include expectation of outcome, participant treatment adherence, circadian rhythm, sleep disorders, and medication status. Depending on the results, additional moderator and mediator analyses will be carried out.

### Ethical Considerations

This clinical trial was approved by the Institutional Ethics Board of Amsterdam University Medical Center on the 10th of March 2022 (2021.0670 - NL78930.029.21) and is registered at clinicaltrials.gov (NCT05331937), on April 15, 2022, last updated on January 15, 2026. All participants give both verbal and written informed consent before completing any study-related procedures. The data will be deidentified and, following the completion of data collection, will be transferred to and stored in a secure data repository with safeguards to protect patient confidentiality. Participants receive a modest reimbursement for participation in the research assessments (not for the treatments), as well as compensation for travel and parking expenses.

### Data Monitoring

Each clinical site oversees the internal quality of study implementation, including data collection, documentation, and study completion. All sites follow a standardized data quality management plan. Before initiating data collection, site principal investigators (site-PIs) and the independent monitor verified that appropriate safety protocols were established at each site. The PI, site-PIs, and other core project members, including the researcher with lived experience (representing the patient foundation), met to review study protocols, emphasizing outcome definitions, study design, serious adverse event (SAE) reporting procedures, informed consent processes, and documentation standards prior to the start of the study. All staff receive training, including a good clinical practice (GCP) course, to carry out assessments, ensuring familiarity with data collection methods, procedural workflows, and SAE reporting requirements. The trial consortium (PI, site-PI’s, core trial researchers, and patient representative) meets monthly to monitor the trial progress.

### Patient Involvement

Prior to the start of data collection, a representative (DVL) from the patient organization (ie, ADF Stichting) was involved in the development of the study design and protocol. Throughout the trial, this representative also participates in monthly consortium meetings to provide ongoing feedback and oversight from a patient perspective. Additionally, patient representatives take part in annual sounding board meetings to provide input aimed at optimizing trial procedures and anticipated implementation of the treatment after study completion.

To enhance transparency and engagement, informational webinars were hosted in collaboration with the patient association (*ADF Stichting*) to inform individuals with OCD about the trial. The research team also receives input from former participants via the ADF Stichting, which is used to improve both the treatment experience and the clarity of trial-related information.

An annual “TETRO Consortium Day” is organized for all participating sites, bringing together rTMS and ERP therapists, researchers, and individuals with OCD to discuss research progress and recent findings from the rTMS, ERP, and OCD fields. During the May 2024 TETRO Consortium Day, a dedicated patient panel discussion was held, providing valuable insights into patient experiences and perspectives that contributed to greater awareness of experience-sensitive aspects of the treatment and study participation among clinicians.

## Results

**Figure 2.**
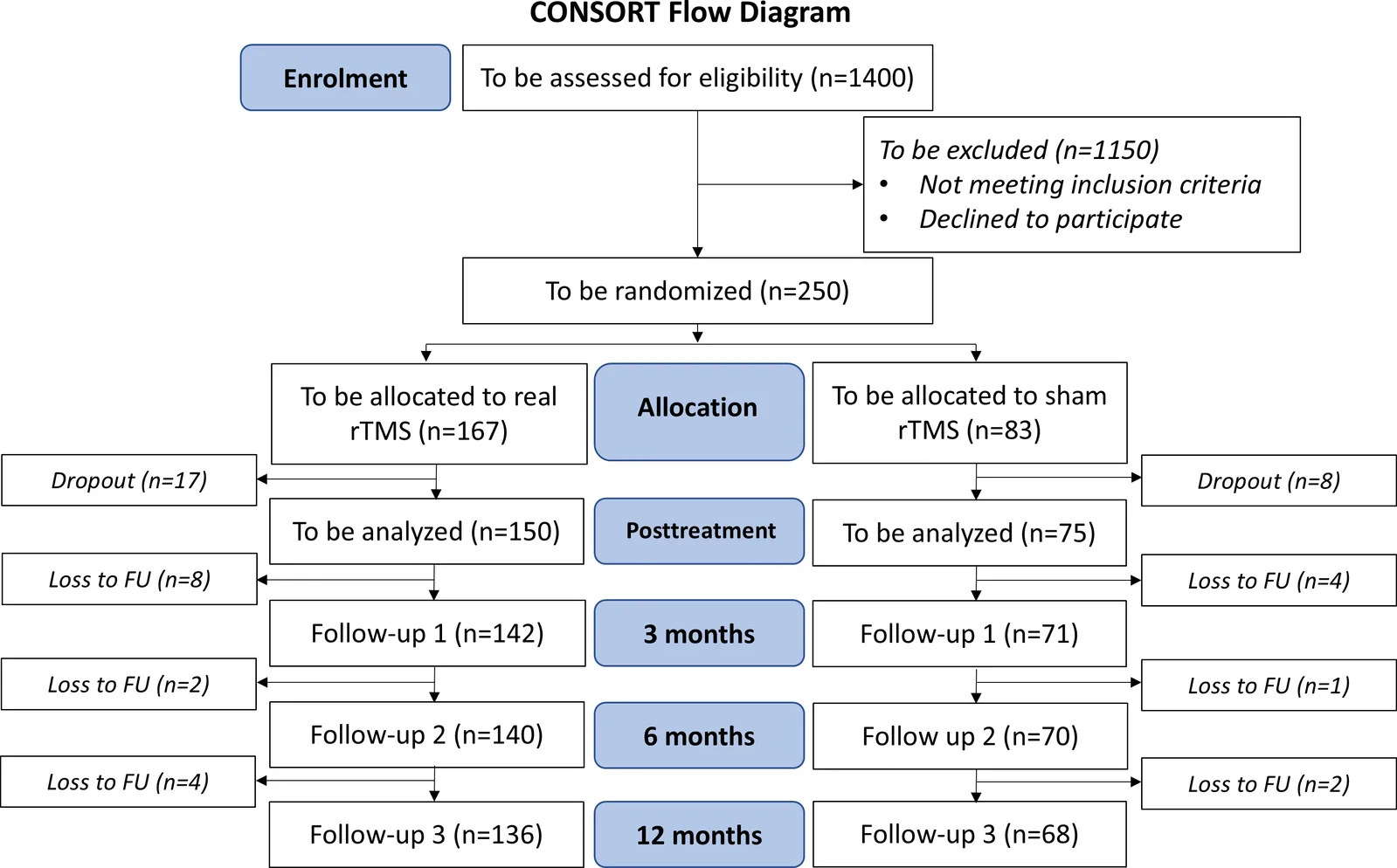
The estimated CONSORT (Consolidated Standards of Reporting Trials) flowchart. The numbers, particularly those for “discontinuation” and “assessed for eligibility,” were predetermined prior to trial initiation. Final figures will be reported in the first analysis following deblinding. FU: follow-up; rTMS: repetitive transcranial magnetic stimulation.

The study received Institutional Review Board approval in March 2022. Recruitment for this RCT started in April 2022. As of February 2026, 201 participants have been enrolled; the final participant is expected to complete their posttreatment assessments by December 2026. Follow-up assessments are expected to be completed by December 2027. A CONSORT (Consolidated Standards of Reporting Trials) flowchart is given in Figure 2.

## Discussion

### Anticipated Findings

This study protocol outlines the first adequately powered multicenter RCT investigating the efficacy and cost-effectiveness of the pre-SMA-rTMS protocol compared to sham-rTMS as adjuvant therapy to ERP in adults with OCD who did not adequately respond to prior first-line treatment. The RCT aims to assess both the clinical efficacy and cost-effectiveness of rTMS targeting the pre-SMA in this population. Alongside the primary focus on efficacy and cost-effectiveness, the study also incorporates several exploratory biological outcome measures designed to shed light on the mechanisms underlying variation in treatment response and short- and long-term treatment-induced neuroplasticity. These measures may ultimately contribute to identifying and developing biomarkers that can inform treatment decisions beyond standard clinical assessments.

It is hypothesized that rTMS combined with ERP will be more efficacious and cost-effective compared to sham-rTMS and ERP. Additionally, we also expect clinical effects (Y-BOCS reduction) in the sham-rTMS group, as both groups receive an intensive format of ERP at expert centers for OCD. The clinical outcomes in the verum group may be comparable to those observed in our previous TIPICCO trial (Clinical Trial ID: NCT03667807 [19], which demonstrated a significant reduction in symptoms (Cohen *d*=1.66, Δ Y-BOCS = −10.836, *P*<.001, 95% CI −12.504 to −9.168) and a 57.4% response rate across 3 different rTMS protocols combined with ERP in a comparable participant group. Participants in that trial received fewer treatment sessions (16 vs 20‐28), shorter ERP sessions (60 vs 90 min), and different TMS protocols (eg, 10 Hz pre-SMA); however, this may allow for improved outcomes in this study.

### Challenges of the Proposed Trial

As of January 2026, we have faced several challenges in the ongoing trial. Coordinating rTMS and ERP, often administered at different locations, has proven logistically complex, requiring careful scheduling among rTMS technicians, therapists, and participants. In Amsterdam, prior experience with the TIPICCO trial enabled the site to begin enrolling the targeted participants concurrently without delay [19]. In contrast, other sites experienced slower start-up times due to difficulties in assembling the full treatment teams, leading to delays in filling participant slots. As a result, the trial has progressed unevenly across sites, with Amsterdam compensating for these delays by enrolling a larger number of participants.

Recruitment also poses challenges, particularly at certain sites. In some cases, this is due to competition from existing intensive ERP programs or a lack of familiarity with rTMS among general practitioners and other referring clinicians. Additionally, some potential participants decline to take part due to the requirement for randomization—especially given that rTMS for OCD is already available in the private (self-paid) health care sector. The intensive treatment schedule (4 sessions per week over 5‐7 wk) is also a barrier for some people, as it can be difficult for participants to manage alongside work or other responsibilities, requiring significant flexibility from both participants and their employers.

### Relevance for Practice

The proposed treatment intends to bridge the gap between conventional therapies (ERP with or without medication) and end-stage invasive strategies such as DBS. Its primary benefit lies in potentially reducing the chronic nature of OCD, which could lead to lower morbidity, improved QoL, enhanced social, educational, and occupational engagement, and reduced health care and productivity-related costs for society.

This multicenter trial involves the use of different rTMS devices across sites, which may introduce some variability due to technical and operational differences. To minimize this, technicians undergo extensive training and are instructed to adhere strictly to standardized operating procedures. However, the inclusion of various machines enhances the external validity and generalizability of the study, reflecting the diversity of equipment used in real-world clinical settings.

The TETRO multicenter study is well-positioned within the national mental health care landscape to enable rapid implementation in secondary and tertiary care settings once the trial concludes. This structure supports broader dissemination, especially important given that the cost of equipment is often a barrier for institutions.

By integrating innovative multimodal neuroimaging and blood-based biomarkers, we aim to identify patient profiles that can help predict who is most likely to respond well to intensive ERP alone or combined rTMS and ERP. Conversely, this approach may also pinpoint individuals with OCD less likely to benefit from ERP or rTMS, who might be candidates for more invasive neuromodulation strategies, such as DBS.

Using the follow-up data, this phenotypical and biological profiling could help detect adults with OCD at increased risk of relapse, who may require additional interventions. Overall, this approach has the potential to enhance personalized treatment strategies for individuals with OCD.

Beyond evaluating the effectiveness of the proposed intervention for OCD, our study advances psychiatric treatment by integrating neuromodulation with behavioral and cognitive therapies. By linking targeted brain stimulation to cognitive-affective processes, this approach supports the development of modular, transdiagnostic treatment protocols, marking a significant step for psychiatric research and clinical care.

### Relevance for Science

This trial uniquely combines a well-powered randomized controlled design, including a 1-year follow-up, with multimodal assessments of neuroplasticity, using both neuroimaging and blood-based biomarkers. This integrative approach is novel and exceptional within the field of OCD research and offers the opportunity to contribute to a more detailed understanding of the neural and molecular changes associated with 2 evidence-based treatments: ERP and rTMS.

Although both interventions have demonstrated effects on brain function, their specific mechanisms of action, and how these may differ, are not yet well understood. In particular, little is known about the potential interaction between rTMS and ERP in modulating neural circuits. By collecting neuroimaging data before and after treatment, along with neuronavigation recordings during treatment and repeated blood sampling, the study will enable investigation of treatment-induced changes at both the neural network and molecular level.

We expect, for instance, to observe changes in cognitive control and emotion regulation networks across conditions, as well as treatment condition-specific effects in the sensorimotor network in the real-rTMS group. These neural findings may correspond with other indicators of neuroplasticity, including expression and methylation of BDNF-related genes and serum BDNF levels. These findings will not only contribute to the field of OCD but will be relevant for many in the booming field of rTMS.

### Dissemination Plan

Findings from this clinical trial will be disseminated through publication in peer-reviewed journals, presentations at national and international scientific conferences, and outreach via social media platforms. All analysis plans will be preregistered on the Open Science Framework (OSF) [65] using the OSF preregistration template, in alignment with open science principles. Study protocols, analysis pipelines, and derived data will be made available upon request after study completion.

The trial’s results, particularly the cost-effectiveness analysis, may inform decision-making by the Dutch National Health Care Institute (Zorginstituut Nederland, ZIN) regarding reimbursement of rTMS for the treatment of OCD. If efficacy is demonstrated, rTMS may additionally be considered at an earlier stage in the treatment pathway according to Dutch guidelines. Due to the study’s integration within established mental health care settings and its geographic distribution across the Netherlands, favorable results could enable relatively swift implementation into clinical practice.

To promote transparency and patient engagement, results will also be shared in accessible, layman’s language with trial participants and other interested individuals with OCD. This will help improve understanding of both the potential benefits and limitations of rTMS as a treatment option.

## Supplementary material

10.2196/94288 Peer Review Report 1 Peer review report 1 from the Promising Care Grant Scheme (ZonMw), National Health Care Institute (Netherlands).

10.2196/94288 Peer Review Report 2 Peer review report 2 from the Promising Care Grant Scheme (ZonMw), National Health Care Institute (Netherlands).

10.2196/94288 Peer Review Report 3 Peer review report 3 from the Promising Care Grant Scheme (ZonMw), National Health Care Institute (Netherlands).

## Abbreviations

(F)MRI: (functional) magnetic resonance imaging
ANCOVA: analysis of covariance
AQ-NL: the autism-spectrum quotient (Dutch version)
BAI: Beck Anxiety Inventory
BDI: Beck Depression Inventory
BDNF: brian-derived neurotrophic factor
BIA: budget impact analysis
CBT: cognitive behavioral therapy
CGI: clinical global impression
CONSORT: Consolidated Standards of Reporting Trials
DALY: disability-adjusted life year
DBS: deep brain stimulation
DLPFC: dorsolateral prefrontal cortex
*DSM-5*: *Diagnostic and Statistical Manual of Mental Disorders, Fifth Edition*
DWI: diffusion weighted image
EEG: electroencephalogram
ERP: exposure therapy with response prevention
GCP: good clinical practice
GWAS: genome-wide association study
HF: high-frequency
HSDQ: Holland Sleep Disorders Questionnaire
ICERs: incremental cost-effectiveness ratios
iMCQ: iMTA Medical Consumption Questionnaire
iPCQ: iMTA productivity cost questionnaire
LF: low-frequency
LMM: linear mixed-model
MAR: missing at random
MDD: major depressive disorder
MINI: Mini-International Neuropsychiatric Interview
MPRAGE: magnetization-prepared rapid acquisition gradient-echo
NDEV: Neuron-Derived Extracellular Vescicle
NOCDA: Netherlands OCD Association
OCD: obsessive-compulsive disorder
PEAS: Patient Exposure and Response Prevention Adherence Scale
PI: principal investigator
Pre-SMA: pre-supplementary motor area
QALYs: quality-adjusted life-years
QoL: quality of life
RCT: randomized controlled trial
RMT: resting motor threshold
rTMS: repetitive transcranial magnetic stimulation
SAE: serious adverse event
SMD: standardized mean difference
SPT: Symptom provocation task
SSRIs: serotonin reuptake inhibitors
VAS: Visual Analog Scale
Y-BOCS: Yale-Brown obsessive compulsive scale
YGTSS: Yale Global Tic Severity Scale
ZIN: Zorginstituut Nederland
