# Supplementary material for: Transcranial Magnetic Stimulation for Patients with Exposure Therapy Resistant Obsessive-Compulsive Disorder (TETRO): Study Protocol for a Multicenter Randomized Controlled Trial
Source: JMIR Res Protoc. 2026 Sep 9;15:e94288. doi: 10.2196/94288 (PMC13557101; doi:10.2196/94288)
Supplement: Supplementary file 1 [file resprot-v15-e94288-s001.pdf]

Subsidieprogramma / Subsidy programme	:	**Subsidieregeling veelbelovende zorg**
Dossiernummer / Dossier number	:	**80-86200-98-20006**
Aanvrager / applicant	:	**Prof. dr. C.H. Polman**
Projecttitel / Project title	:	**Transcranial magnetic stimulation (TMS) for exposure treatment-resistant obsessive-compulsive disorder (OCD) - TETRO, a multi-center randomized clinical trial**
Beoordelingscode / Assessment code	:	**B.2020.022E0**

1. Objective

Legenda: G (Good), F (Fair), P (Poor)

1.1 Research question and hypothesis

G	F	P
	X	

Please consider the following:

- is the research question clear and concrete?
- is the hypothesis clear and verifiable?
- is the hypothesis consistent with the research question?
- are there (other) concerns with regards to the research question or hypothesis?

Please indicate the strengths and weaknesses.

Answer:

The research question is clear and concrete. A strength is that the focus is on clinical important effects and not only more efficacy in general. The hypothesis is too broad "treatment will reduce the OCD burden significantly" and is therefore not verifiable. It is unclear how OCD burden is defined and what is considered a significant decrease in burden.

The rationale for the choice to add rTMS to ERP is not well explained. All patients failed on ERP and now they will get the treatment again. Please provide evidence why rTMS could help to let ERP succeed?

2. Strategy

Legenda: G (Good), F (Fair), P (Poor)

2.1 Adequacy of the clinical study design

G	F	P
X		

The adequacy of the chosen study design is an important aspect. The goal of the research is to provide a reliable estimate of the effectiveness and cost-effectiveness of the new treatment compared to the standard of care (in the Netherlands). Applicants should have provided a rational for all the choices they have made.

Please consider the following:

- is the design adequately motivated in the grant proposal? is the design appropriate for the research question and is it adequate to answer the research question?

* For Dutch reviewers: does the control group consist of the Dutch standard of care? Please note that the standard of care could be best supportive care.

- if there is no randomization: do you believe randomization is feasible?
- are there (other) concerns with regard to the study design?

Please indicate the strengths and weaknesses.

Answer:

A multicenter randomized controlled trial is an adequate study design to answer the research question. A strength of the current study is that it is possible to use sham-rTMS, which makes it possible to blind patients and outcome assessors for the treatment received.

For the cost-effectiveness study is not completely clear why besides the trial-based economic evaluation, a modelling study will be performed. Modelling studies are usually performed when only intermediate parameters are measured in the trial. A model then extrapolates these effects to long-term outcomes. Is the effect on QALYs expected to increase over time? Or is this mainly based on the idea that treatment in year one, will also reduce healthcare and productivity costs in years thereafter, while initial costs of treatment are not annual. Modelling requires quite some assumptions on the maintenance of the effect after the initial 5-7 weeks.

According to guidelines standard of care treatment for OCD in the Netherlands indeed exists of pharmacological therapy and/or psychological interventions, such as ERP and cognitive therapy. ERP is first choice, because of its effectiveness and evidence on long-term effects. So yes, ERP with or without medications seems a good comparator in the current study.

2.2 Methods of the clinical study

G	F	P
X		

The grant proposal should include information on the eligibility criteria, outcomes, follow-up and sample size & statistical analyses.

Please consider the following:

- *Topic eligibility criteria*: 1) are all relevant eligibility criteria included? If the answer to this question is no, which criteria should be added or deleted?
- *Topic outcomes*: 1) is the primary outcome(s) the most important outcome(s) to assess the relative effectiveness? If the answer is no, which (patient relevant) outcome would you consider a better choice as primary outcome? 2) if a surrogate or intermediate outcome is chosen as primary outcome, has the applicant shown adequately that there is a (verifiable) relationship with a hard outcome (such as death)? 3) are all (important) outcomes to assess the benefit and harm of the new intervention selected?
- *Topic follow-up*: 1) is the follow-up sufficient to assess the benefits and harms of the new intervention compared to the standard of care? In the answer is no, what would be the optimal follow-up time to assesses the relative effectiveness?
- *Topic sample size & statistical analyses*: 1) is the minimal clinically important difference (MCID) clinically relevant? 2) is the sample size calculation sufficient and in line with the systematic review or other research? 3) is the description and quality of the of statistical methods sufficient? 4) is there a need for additional (subgroup) analyses?
- are there (other) concerns with regards to the chose methods or the rational for the choices?

Please indicate the strengths and weaknesses.

Answer:

In and exclusion criteria for patients are extensively described. A strength of the current study is that patients with comorbid conditions are included, as long as OCD is the primary diagnosis.

The primary outcome is the Y-BOCS which measured the severity of OCD symptomes. This is a relevant outcome from a patient perspective. For the primary outcome the change in baseline to end of treatment (week 5/6/7) score is considered. The outcomes at 12 months are however more relevant to show whether the effects will also be maintained over time. This is however difficult to assess with the current study design, because the trial uses a natural follow-up up to 12 months, in which patients could get other treatments. Secondary outcomes seems all relevant as well.

Follow-up duration of 12 months after an initial treatment period of 5-7 weeks is very relevant to assess whether the effects are maintained, which will however, be difficult to assess (see above).

Multilevel linear mixed-model (LMM) regression analysis is an appropriate analysis to analyze repeated individual patient data, while taken into account that patients come from different treatment centers (clusters).

The sample size calculations uses an expected difference in Y-BOCS of 0.4, which corresponds to four points decrease and can be considered a clinical relevant improvement. However, the meta-analysis performed in the systematic review showed a treatment effect of 0.5 for rTMS versus control as monotherapy i.e. not in addition to ERP. In the current study rTMS is added to ERP and compared to ERP alone, which probably reduces the room for improvement for rTMS. In that perspective a estimate of treatment effect of 0.4 might not be as conservative as the authors suggest.

There is no need for additional subgroups as the authors already mentioned all relevant ones.

2.3 Feasibility

G	F	P
	X	

In the grant proposal the applicants should demonstrate that the project is feasible within the proposed timeframe.

Please consider the following:

- are all components needed to assess the feasibility (such as incidence/prevalence, number of patients seen in participating centers, percentage of patients willing to participate, time for recruitment, time for analyses, etc.) included in the grant proposal? And based on this data do you believe the timetable realistic?
- is the number of participating centers sufficient?
- are there (other) concerns with regards to the feasibility?

Please indicate the strengths and weaknesses.

Answer:

The feasibility in terms of total number of patients to include seems realistic, however not the distribution over the different study centers as proposed.

Based on the annual number of referrals in Amsterdam and Nijmegen, it should not be a problem to have 60 patients included in the trial. However, 60 patients for Maastricht seems already more difficult. Assuming a 4 year inclusion period and 50% eligibility this would mean that about 50% of the eligible patients need to participate.

For the two neuroCare sites, this percentage is even higher. Over a 4 year period, about 40 patients would be eligible of which 35 need to be included. That seems not realistic.

2.4 Cost-effectiveness analysis

G	F	P
X		

The purpose of the cost-effectiveness analysis (CEA) is to assess the proposed gain in health care efficiency of the (new) intervention(s) compared to the usual care provided in the Netherlands.

Please consider the following:

- whether all elements of PICOT (patients, intervention, comparator(s), outcomes, time horizon) are clearly stated in the cost-effectiveness analysis
- whether PICOT is consistent with the research questions
- whether the CEA is well designed
- whether the methods of data collection are appropriate
- whether all relevant cost categories (i.e. direct- and indirect medical/ non-medical costs) are included?
- to your knowledge is the CEA consistent with the Dutch guideline for health economic evaluations? See report Cost Effectiveness in Practice: https://english.zorginstituutnederland.nl/publications/reports/2015/06/16/cost-effectiveness-in-practice

Please indicate the strengths and weaknesses.

Answer:

The cost-effectiveness analysis is well designed. The study using a societal perspective which is according to guidelines and includes all required costs categories (healthcare costs, productivity costs, informal care costs).
The data will be collected with commonly used valid questionnaires.
The cost-effectiveness study uses a one-year time horizon. Due to the natural follow-up after week 7 it might be difficult to assess what the gain in QALYs is due to rTMS, because patients can use other treatments between week 7 and 12 months.

As mentioned before, the relevance of using a modelling approach besides the trial-based economic evaluation is not explained.

2.5 Budget impact analysis

G	F	P
	X	

The purpose of the budget impact analysis (BIA) is to assess the financial consequences of dissemination of the (new) intervention(s). Information obtained from the BIA can be used for policy decisions on a national, regional and/or local level. Various perspectives can be taken into account; the government and insurance perspectives must always be considered.

Please consider the following:

- is the BIA well-designed in terms of perspectives, scenarios, population, time horizon, etc.?
- are all relevant cost categories (i.e. direct medical costs) included?
- Is the BIA consistent with the Dutch guideline (Chapter 5) for health economic evaluations?

Please indicate the strengths and weaknesses.

Answer:

The proposal mentions that effects and costs of the trial will be combined with prevalence and incidence data, which is correct. Although it is not clear how effects will be used as budget impact is about costs.

The proposal does not mention the uptake of the new treatment (ERP + rTMS) and how this will be estimated. This is one of the most relevant parameters in a BIA.

2.6 Systematic review

G	F	P
X		

Please consider the following:

- is the selection of search terms sufficient?
- are all important studies included which assessed the effectiveness? If no, which relevant article(s) are missing?
- are all (important) databases included in the search?
- are the conclusions of the systematic review with regards to the estimate of the relative effectiveness justified?
- are there additional weaknesses with regards to the systematic review?

Please indicate the strengths and weaknesses.

Answer:

The quality of the systematic review was ok. Relevant databases were searched and selection of search terms seems sufficient.

The overall conclusion of the review was correct. However, subgroup analyses for simulation protocol showed three options to be effective. PreSMA is recommended in the conclusion because this is best supported by evidence. Indeed results for this subgroup were based on 6 studies. However, the other two protocols seems substantially more effective, but based on less studies (3 and 2, respectively). The conclusion on simulation protocol can therefore be debated.

3. Project group

Legenda: G (Good), F (Fair), P (Poor)

3.1 Project group

G	F	P
X		

Please consider the following:

- relevant expertise and disciplines; Is expertise missing? If yes, what is missing?
- familiarity with research area
- prior activities and products.

Please indicate the strengths and weaknesses of the project group.

Answer:

All project members have expertise in the area of OCD and/or rTMS, which is reflected in several publications on these topics. The project leader has extensive experience with the topic, as she is currently performing a proof-of-concept trial for rTMS in OCD. Project members participate in relevant foundations, societies and expertise groups.

Health economic and statistical expertise are also included sufficiently.

4. Overall quality assessment

Legenda: VG (Very good), G (Good), S (Sufficient), F (Fair), P (Poor)

4.1 Overall quality assessment

VG	G	S	F	P
	X			

Give the overall quality assessment of the grant application (regarding points 1-3). Please summarize the crucial strengths and weaknesses of the grant application.

The study is well designed and the proposed statistical methods seem correct. Outcomes included are relevant also from a patient perspective.

The rationale and evidence why ERP would work in combination with rTMS in a patient population that failed on ERP before is not provided.

The feasibility in terms of the overall number of patients to recruit seems realistic, but not in terms of the distribution over the different centers.

Project team has a lot of experience on the topic and has close connections to clinical practice.

5. Additional value to current knowledge

Legenda: G (Good), F (Fair), P (Poor)

5.1 Additional value to current knowledge

G	F	P
X		

Consider:

- will this project yield new information to determine the relative effectiveness?;
- will this project the project yield new information compared to ongoing projects?

Answer:

The patient population is a large group of patients that failed on the currently most important treatment option. Besides this, there are not much other treatment options for this group, except for antidepressants and antipsychotics which have side effects and are not preferred by patients.
rTMS could offer them an non-pharmacological alternative.

The project yields new information, because rTMS has been studied before as monotherapy, while the current trial will study the impact of rTMS added to ERP. However, as mentioned before the rationale why ERP in combination with rTMS would work in patients who failed on ERP before is missing. The applicants mention that "they expect better effects rTMS and ERP are combined.

The proposals mentions several ongoing studies on the topic: proof-of-concept trial by prof van den Heuvel, and "we expect better effects when rTMS is used as adjuvant therapy to ERP (as is currently also the practice in ongoing experimental rTMS studies". It is unclear what the current study would add to these ongoing studies.

rTMS has been proven to be effective for depression and is therefore reimbursed for this patient group. Reimbursement decisions for OCD should be evidence-based to avoid spending healthcare budgets in an inefficient way. This trial could help to provide this evidence.
