# Supplementary material for: Transcranial Magnetic Stimulation for Patients with Exposure Therapy Resistant Obsessive-Compulsive Disorder (TETRO): Study Protocol for a Multicenter Randomized Controlled Trial
Source: JMIR Res Protoc. 2026 Sep 9;15:e94288. doi: 10.2196/94288 (PMC13557101; doi:10.2196/94288)
Supplement: Supplementary file 2 [file resprot-v15-e94288-s002.pdf]

Subsidieprogramma / Subsidy programme	:	**Subsidieregeling veelbelovende zorg**
Dossiernummer / Dossier number	:	**80-86200-98-20006**
Aanvrager / applicant	:	**Prof. dr. C.H. Polman**
Projecttitel / Project title	:	**Transcranial magnetic stimulation (TMS) for exposure treatment-resistant obsessive-compulsive disorder (OCD) - TETRO, a multi-center randomized clinical trial**
Beoordelingscode / Assessment code	:	**B.2020.022E1**

1. Objective

Legenda: G (Good), F (Fair), P (Poor)

1.1 Research question and hypothesis

G	F	P
X		

Please consider the following:

- is the research question clear and concrete?
- is the hypothesis clear and verifiable?
- is the hypothesis consistent with the research question?
- are there (other) concerns with regards to the research question or hypothesis?

Please indicate the strengths and weaknesses.

Answer:

Superb proposal that aims at investigating the therapeutic potential of TMS as an adjunct to psychotherapy for OCD. The hypotheses are clear, verifiable and consistent with the research question, such that I have no concerns regarding this part of the proposal.
Strengths
- Stratification along nonresponse to psychotherapy (20 sessions) rather than pharmacotherapy
- Target population: Treatment-resistant patients with OCD (50%) where DBS is no option yet.
- Adequate power (N = 250 patients)
- Each intervention consists of TMS followed by psychotherapy
Weaknesses
- Heterogeneity in comorbidities as well as medications may confound the outcomes
- Potentially high dropout-rate due to high-frequency treatment (4 days/week over 5-7 weeks)

2. Strategy

Legenda: G (Good), F (Fair), P (Poor)

2.1 Adequacy of the clinical study design

G	F	P
X		

The adequacy of the chosen study design is an important aspect. The goal of the research is to provide a reliable estimate of the effectiveness and cost-effectiveness of the new treatment compared to the standard of care (in the Netherlands). Applicants should have provided a rational for all the choices they have made.

Please consider the following:

- is the design adequately motivated in the grant proposal? is the design appropriate for the research question and is it adequate to answer the research question?

* For Dutch reviewers: does the control group consist of the Dutch standard of care? Please note that the standard of care could be best supportive care.

- if there is no randomization: do you believe randomization is feasible?
- are there (other) concerns with regard to the study design?

Please indicate the strengths and weaknesses.

Answer:

Excellent clinical study design (2-arm RCT with an active adjuvant rTMS treatment to daily ERP) that includes a control condition (Sham TMS) and pre-versus-post-treatment standardized mean difference (SMD) in severity of OCD as primary outcome. No weaknesses.

2.2 Methods of the clinical study

G	F	P
X		

The grant proposal should include information on the eligibility criteria, outcomes, follow-up and sample size & statistical analyses.

Please consider the following:

- *Topic eligibility criteria*: 1) are all relevant eligibility criteria included? If the answer to this question is no, which criteria should be added or deleted?
- *Topic outcomes*: 1) is the primary outcome(s) the most important outcome(s) to assess the relative effectiveness? If the answer is no, which (patient relevant) outcome would you consider a better choice as primary outcome? 2) if a surrogate or intermediate outcome is chosen as primary outcome, has the applicant shown adequately that there is a (verifiable) relationship with a hard outcome (such as death)? 3) are all (important) outcomes to assess the benefit and harm of the new intervention selected?
- *Topic follow-up*: 1) is the follow-up sufficient to assess the benefits and harms of the new intervention compared to the standard of care? In the answer is no, what would be the optimal follow-up time to assesses the relative effectiveness?
- *Topic sample size & statistical analyses*: 1) is the minimal clinically important difference (MCID) clinically relevant? 2) is the sample size calculation sufficient and in line with the systematic review or other research? 3) is the description and quality of the of statistical methods sufficient? 4) is there a need for additional (subgroup) analyses?
- are there (other) concerns with regards to the chose methods or the rational for the choices?

Please indicate the strengths and weaknesses.

Answer:

Outcome variables, sample size and statistical analyses have been adequately chosen. No weaknesses.

2.3 Feasibility

G	F	P
X		

In the grant proposal the applicants should demonstrate that the project is feasible within the proposed timeframe.

Please consider the following:

- are all components needed to assess the feasibility (such as incidence/prevalence, number of patients seen in participating centers, percentage of patients willing to participate, time for recruitment, time for analyses, etc.) included in the grant proposal? And based on this data do you believe the timetable realistic?
- is the number of participating centers sufficient?
- are there (other) concerns with regards to the feasibility?

Please indicate the strengths and weaknesses.

Answer:

Given that this is a multi-center trial, and given that OCD is difficult to treat in more than half of the cases, recruitment of subjects is feasible. No weaknesses.

2.4 Cost-effectiveness analysis

G	F	P
	X	

The purpose of the cost-effectiveness analysis (CEA) is to assess the proposed gain in health care efficiency of the (new) intervention(s) compared to the usual care provided in the Netherlands.

Please consider the following:

- whether all elements of PICOT (patients, intervention, comparator(s), outcomes, time horizon) are clearly stated in the cost-effectiveness analysis
- whether PICOT is consistent with the research questions
- whether the CEA is well designed
- whether the methods of data collection are appropriate
- whether all relevant cost categories (i.e. direct- and indirect medical/ non-medical costs) are included?
- to your knowledge is the CEA consistent with the Dutch guideline for health economic evaluations? See report Cost Effectiveness in Practice: https://english.zorginstituutnederland.nl/publications/reports/2015/06/16/cost-effectiveness-in-practice

Please indicate the strengths and weaknesses.

Answer:

The proposal states that both a cost-effectiveness analysis (CEA) and a cost-utility analysis (CUA) will be performed from a societal and healthcare perspective according to Dutch guidelines (Zorginstituut Nederland. Richtlijn voor het uitvoeren van economische
evaluaties in de gezondheidszorg. Diemen; 2016) with a time horizon of 12 months.

2.5 Budget impact analysis

G	F	P
	X	

The purpose of the budget impact analysis (BIA) is to assess the financial consequences of dissemination of the (new) intervention(s). Information obtained from the BIA can be used for policy decisions on a national, regional and/or local level. Various perspectives can be taken into account; the government and insurance perspectives must always be considered.

Please consider the following:

- is the BIA well-designed in terms of perspectives, scenarios, population, time horizon, etc.?
- are all relevant cost categories (i.e. direct medical costs) included?
- Is the BIA consistent with the Dutch guideline (Chapter 5) for health economic evaluations?

Please indicate the strengths and weaknesses.

Answer:

The proposal states that a budget impact analysis (BIA) will be conducted from the perspective of health-care decision makers according to the Dutch
guidelines (Zorginstituut Nederland, 2016) and the recommendations from Sullivan et al. (2014).

2.6 Systematic review

G	F	P
	X	

Please consider the following:

- is the selection of search terms sufficient?
- are all important studies included which assessed the effectiveness? If no, which relevant article(s) are missing?
- are all (important) databases included in the search?
- are the conclusions of the systematic review with regards to the estimate of the relative effectiveness justified?
- are there additional weaknesses with regards to the systematic review?

Please indicate the strengths and weaknesses.

Answer:

Excellent systematic review that leaves no open questions.

3. Project group

Legenda: G (Good), F (Fair), P (Poor)

3.1 Project group

G	F	P
X		

Please consider the following:

- relevant expertise and disciplines; Is expertise missing? If yes, what is missing?
- familiarity with research area
- prior activities and products.

Please indicate the strengths and weaknesses of the project group.

Answer:

Superb project group with excellent clinician scientists who are leaders in their respective fields.

4. Overall quality assessment

Legenda: VG (Very good), G (Good), S (Sufficient), F (Fair), P (Poor)

4.1 Overall quality assessment

VG	G	S	F	P
X				

Give the overall quality assessment of the grant application (regarding points 1-3). Please summarize the crucial strengths and weaknesses of the grant application.

A great multi-center project that combines an excellent research question with superb expertise on behalf of the members of the project group.

5. Additional value to current knowledge

Legenda: G (Good), F (Fair), P (Poor)

5.1 Additional value to current knowledge

G	F	P
X		

Consider:

- will this project yield new information to determine the relative effectiveness?;
- will this project the project yield new information compared to ongoing projects?

Answer:

The proposed treatment intends to fill the gap between standard therapies (ERP with / without medication) and end-stage invasive strategies. The greatest added value will be the reduction in disease chronicity, resulting in decreased morbidity,
improved quality of life, increased social and education/occupational participation and decreased healthcare and lost productivity costs for the society.
