# Supplementary material for: Transcranial Magnetic Stimulation for Patients with Exposure Therapy Resistant Obsessive-Compulsive Disorder (TETRO): Study Protocol for a Multicenter Randomized Controlled Trial
Source: JMIR Res Protoc. 2026 Sep 9;15:e94288. doi: 10.2196/94288 (PMC13557101; doi:10.2196/94288)
Supplement: Supplementary file 3 [file resprot-v15-e94288-s003.pdf]

Subsidieprogramma / Subsidy programme	:	**Subsidieregeling veelbelovende zorg**
Dossiernummer / Dossier number	:	**80-86200-98-20006**
Aanvrager / applicant	:	**Prof. dr. C.H. Polman**
Projecttitel / Project title	:	**Transcranial magnetic stimulation (TMS) for exposure treatment-resistant obsessive-compulsive disorder (OCD) - TETRO, a multi-center randomized clinical trial**
Beoordelingscode / Assessment code	:	**B.2020.022E2**

1. Objective

Legenda: G (Good), F (Fair), P (Poor)

1.1 Research question and hypothesis

G	F	P
X		

Please consider the following:

- is the research question clear and concrete?
- is the hypothesis clear and verifiable?
- is the hypothesis consistent with the research question?
- are there (other) concerns with regards to the research question or hypothesis?

Please indicate the strengths and weaknesses.

Answer:

The research question is clear. Active Sham rTMS is a good control. However it will be difficult to tease apart effects of rTMS from ERP in the current design. ERP done properly will be robustly effective. The question is if this is the best augmentation approach to sit between outpatient approaches and DBS. One could argue that it is; other positions might suggest more intensive treatment approaches such as those offered in this study. But, this project will advance the field in ways including the research question, as well as other forms of data collection (e.g., imaging).

2. Strategy

Legenda: G (Good), F (Fair), P (Poor)

2.1 Adequacy of the clinical study design

G	F	P
	X	

The adequacy of the chosen study design is an important aspect. The goal of the research is to provide a reliable estimate of the effectiveness and cost-effectiveness of the new treatment compared to the standard of care (in the Netherlands). Applicants should have provided a rational for all the choices they have made.

Please consider the following:

- is the design adequately motivated in the grant proposal? is the design appropriate for the research question and is it adequate to answer the research question?

* For Dutch reviewers: does the control group consist of the Dutch standard of care? Please note that the standard of care could be best supportive care.

- if there is no randomization: do you believe randomization is feasible?
- are there (other) concerns with regard to the study design?

Please indicate the strengths and weaknesses.

Answer:

The basis that 50% are helped with ERP is incorrect. No study of ERP has ever found only a 50% response rate. The rate is about 75% responders about 40% remitters. Precision in definition is needed.

Further, the applicants cite meta-analyses by others that show effect of rTMS to be around .79, but their own

estimates are much lower. It sets up a bit of an inaccurate argument about the potential of rTMS benefits. The question is if this is the best augmentation approach to sit between outpatient approaches and DBS. One could argue that it is; other positions might suggest more intensive treatment approaches such as those offered in this study. But, this project will advance the field in ways including the research question, as well as other forms of data collection (e.g., imaging).

The design will make it difficult to tease out effects of rTMS from ERP. That is, it may be difficult to separate two very active interventional arms at this point. However, this is an empirical question.

ERP provided should make randomization and recruitment feasible.

The adaptive design of rTMS is confusing and introduces confound of differential treatment. At this stage it would be ideal to standardize dose.

2.2 Methods of the clinical study

G	F	P
X		

The grant proposal should include information on the eligibility criteria, outcomes, follow-up and sample size & statistical analyses.

Please consider the following:

- *Topic eligibility criteria*: 1) are all relevant eligibility criteria included? If the answer to this question is no, which criteria should be added or deleted?
- *Topic outcomes*: 1) is the primary outcome(s) the most important outcome(s) to assess the relative effectiveness? If the answer is no, which (patient relevant) outcome would you consider a better choice as primary outcome? 2) if a surrogate or intermediate outcome is chosen as primary outcome, has the applicant shown adequately that there is a (verifiable) relationship with a hard outcome (such as death)? 3) are all (important) outcomes to assess the benefit and harm of the new intervention selected?
- *Topic follow-up*: 1) is the follow-up sufficient to assess the benefits and harms of the new intervention compared to the standard of care? In the answer is no, what would be the optimal follow-up time to assesses the relative effectiveness?
- *Topic sample size & statistical analyses*: 1) is the minimal clinically important difference (MCID) clinically relevant? 2) is the sample size calculation sufficient and in line with the systematic review or other research? 3) is the description and quality of the of statistical methods sufficient? 4) is there a need for additional (subgroup) analyses?
- are there (other) concerns with regards to the chose methods or the rational for the choices?

Please indicate the strengths and weaknesses.

Answer:

Methodology is sound overall.

Limited attention to ERP and rater fidelity which is critical.

Multiple therapists for ERP is fine but more attention needed to protocol since this is a large component of the study.

Rating battery is acceptable but not innovative.

YBOCS > 16 seems rather low and likely to introduce placebo responders.

2.3 Feasibility

G	F	P
X		

In the grant proposal the applicants should demonstrate that the project is feasible within the proposed timeframe.

Please consider the following:

- are all components needed to assess the feasibility (such as incidence/prevalence, number of patients seen in participating centers, percentage of patients willing to participate, time for recruitment, time for analyses, etc.) included in the grant proposal? And based on this data do you believe the timetable realistic?

- is the number of participating centers sufficient?
- are there (other) concerns with regards to the feasibility?

Please indicate the strengths and weaknesses.

Answer:

No concerns about feasibility.
Addition of ERP should facilitate recruitment.

2.4 Cost-effectiveness analysis

G	F	P
X		

The purpose of the cost-effectiveness analysis (CEA) is to assess the proposed gain in health care efficiency of the (new) intervention(s) compared to the usual care provided in the Netherlands.

Please consider the following:

- whether all elements of PICOT (patients, intervention, comparator(s), outcomes, time horizon) are clearly stated in the cost-effectiveness analysis
- whether PICOT is consistent with the research questions
- whether the CEA is well designed
- whether the methods of data collection are appropriate
- whether all relevant cost categories (i.e. direct- and indirect medical/ non-medical costs) are included?
- to your knowledge is the CEA consistent with the Dutch guideline for health economic evaluations? See report Cost Effectiveness in Practice: https://english.zorginstituutnederland.nl/publications/reports/2015/06/16/cost-effectiveness-in-practice

Please indicate the strengths and weaknesses.

Answer:

No concerns noted.

2.5 Budget impact analysis

G	F	P
X		

The purpose of the budget impact analysis (BIA) is to assess the financial consequences of dissemination of the (new) intervention(s). Information obtained from the BIA can be used for policy decisions on a national, regional and/or local level. Various perspectives can be taken into account; the government and insurance perspectives must always be considered.

Please consider the following:

- is the BIA well-designed in terms of perspectives, scenarios, population, time horizon, etc.?
- are all relevant cost categories (i.e. direct medical costs) included?
- Is the BIA consistent with the Dutch guideline (Chapter 5) for health economic evaluations?

Please indicate the strengths and weaknesses.

Answer:

No concerns noted. All indicated elements are included.

2.6 Systematic review

G	F	P
X		

Please consider the following:

- is the selection of search terms sufficient?
- are all important studies included which assessed the effectiveness? If no, which relevant article(s) are missing?
- are all (important) databases included in the search?

- are the conclusions of the systematic review with regards to the estimate of the relative effectiveness justified?
- are there additional weaknesses with regards to the systematic review?

Please indicate the strengths and weaknesses.

Answer:

The review is inclusive and appropriate.

3. Project group

Legenda: G (Good), F (Fair), P (Poor)

3.1 Project group

G	F	P
	X	

Please consider the following:

- relevant expertise and disciplines; Is expertise missing? If yes, what is missing?
- familiarity with research area
- prior activities and products.

Please indicate the strengths and weaknesses of the project group.

Answer:

The team is very strong.

Dr. van den Heuvel is a leader and OCD expert.

There is strong expertise in brain stimulation on the team.

There is appropriate statistical expertise.

There is no senior ERP expert identified on the team.

4. Overall quality assessment

Legenda: VG (Very good), G (Good), S (Sufficient), F (Fair), P (Poor)

4.1 Overall quality assessment

VG	G	S	F	P
	X			

Give the overall quality assessment of the grant application (regarding points 1-3). Please summarize the crucial strengths and weaknesses of the grant application.

This is a very strong project by a strong research group. The project is feasible and likely to have a modest impact on the field depending on results. Enthusiasm is reduced by several factors:
- rTMS demonstrates modest effects relative to sham as the applicants' meta shows. The question is if this is the best augmentation approach to sit between outpatient approaches and DBS.
-true effects of rTMS and ability to distinguish effects of rTMS + ERP over ERP alone.
-Inclusion criteria of YBOCS >15.
- Nature of ERP provision/supervision/fidelity.
- Modest innovation which could be improved with discussion of hypothesized neural predictors with baseline imaging.
-Justification for 10% attrition over the course of the study (not just acute treatment).

5. Additional value to current knowledge

Legenda: G (Good), F (Fair), P (Poor)

5.1 Additional value to current knowledge

G	F	P
X		

Consider:

- will this project yield new information to determine the relative effectiveness?;
- will this project the project yield new information compared to ongoing projects?

Answer:

This study has the potential to impact the field. rTMS demonstrates modest effects relative to sham as the applicants' meta shows. The question is if this is the best augmentation approach to sit between outpatient approaches and DBS. The questions are relevant and the team is outstanding. This project will inform if rTMS+ERP beats ERP alone, which is important.
